# Supplementary material for: Nitrate leaching and its implication for Fe and As mobility in a Southeast Asian aquifer
Source: FEMS Microbiol Ecol. 2023 Mar 14;99(4):fiad025. doi: 10.1093/femsec/fiad025 (PMC10038221; doi:10.1093/femsec/fiad025)
Supplement: fiad025_Supplemental_File [file fiad025_supplemental_file.docx]

**Supplementary Materials**

**Calculation of the total amount of ^13^CO_2_ and ^15^N_2_O**

**∑^13^CO_2_ = ^13^CO_2(g)_ [1 + kRT V_liquid_/V_gas_ (1+ K_Z_/[H^+^])]** Equation S1

Where:

1) ∑^13^CO_2_ is the total amount of ^13^CO_2_ in the bottle

2) ^13^CO_2(g)_ is the amount of ^13^CO_2_ in the gas phase (headspace) in mmol.

3) k is the solubility coefficient of CO_2_, which is 3.3x10^-4^ mol/m^3^ Pa.

4) R is the universal gas constant, from the ideal gas law, which is 8.314 J mol^-1^ K^-1^.

5) T is the Kelvin temperature of the incubation condition. 4ºC = 277.15 K, RT is ~22 ºC = 295.15 K

6) V_liquid_ and V_gas_ are the volumes of the liquid and gas phases (in mL).

7) K_Z_ is the dissociation constant of the first step of carbonic acid dissociation. That is K_a1_, which you get from p*K_1_*, which you have to calculate (see below).

8) [H^+^] is the molar concentration of H^+^

The equation for calculating N_2_O is very similar to the equation S1

**∑^14^N_2_O = ^14^N_2_O _(g)_ [1 + kRT V_liquid_/V_gas_ ]** Equation S2

1) ∑^14^ N_2_O is the total amount of ^14^ N_2_O in the bottle

2) ^14^ N_2_O _(g)_ is the amount of ^14^ N_2_O in the gas phase (headspace) in mmol.

3) k is the solubility coefficient of CO_2_, which is 2.4*10^-4^mol/m^3^ Pa.

4) R is the universal gas constant, from the ideal gas law, which is 8.314 J mol^-1^ K^-1^.

5) T is the Kelvin temperature of the incubation condition. 4ºC = 277.15 K, RT is ~22 ºC = 295.15 K

6) V_liquid_ and V_gas_ are the volumes of the liquid and gas phases (in mL).

7) The K_Z_ of N_2_O is 0 because it does not react with water.


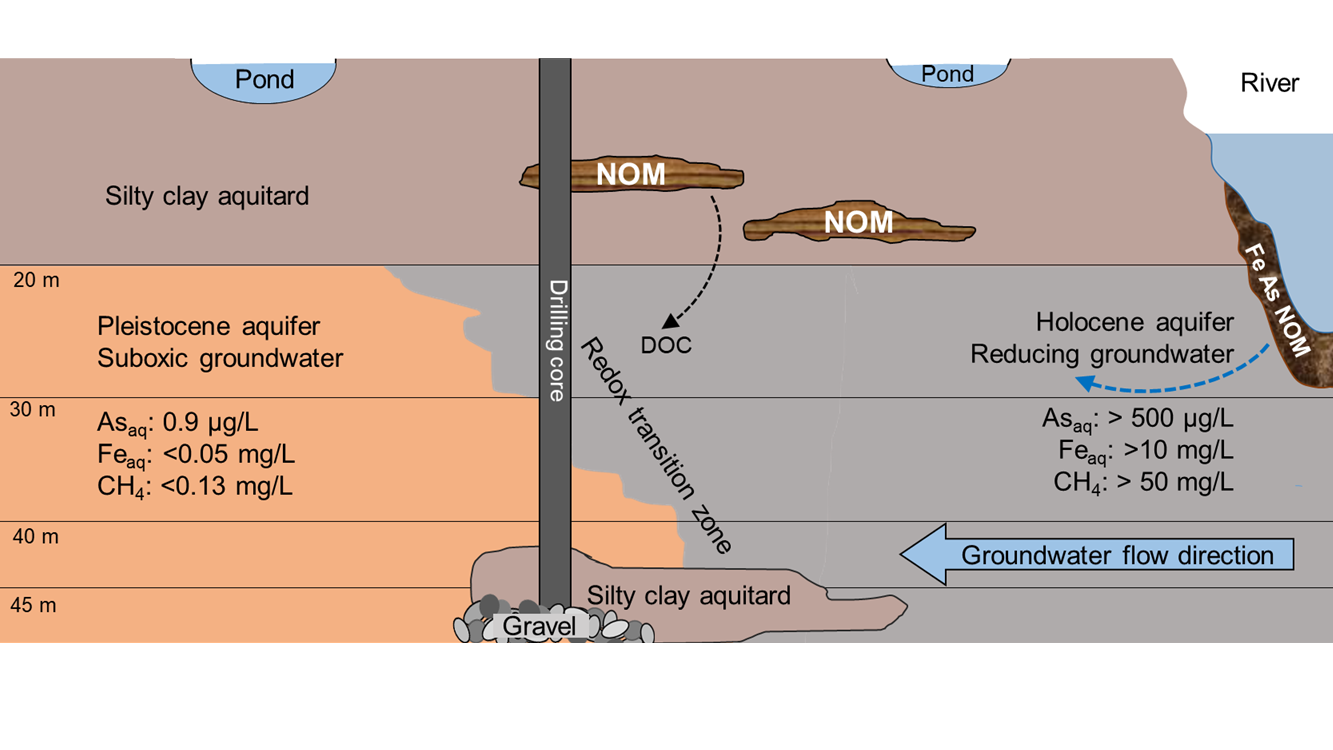


**Fig. S1**. Two-dimensional cross-section of Van Phuc aquifer with the redox transition zone (RTZ) which divides gray As-contaminated aquifer of Holocene origin and Pleistocene non-contaminated aquifer consisting of yellow-brown sediments. Modified from Glodowska et al. 2021.


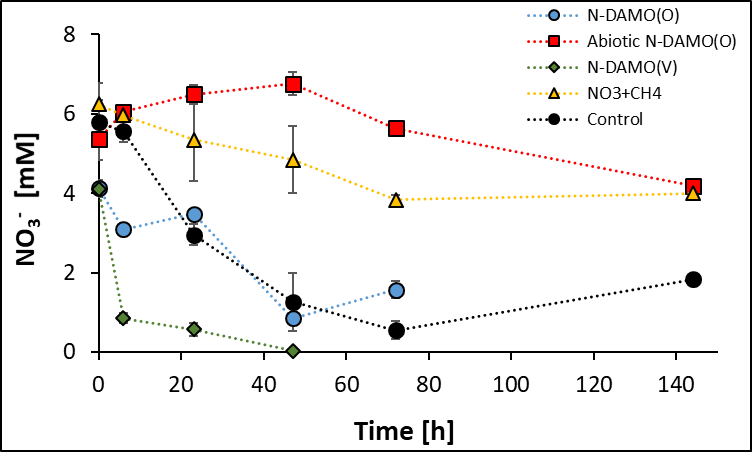


**Fig. S2 Changes of NO_3_^-^ concentration in five treatments at second NO_3_^-^ injection after 65 days of incubations**

Please note that the error bar in this figure represents the standard error of three measurements. One microcosm from each of the five treatments was used for this assay.

After the second injection, the NO_3_^-^ concentration in the CH_4_and NO_3_^-^ amended treatment only decreased by 2.24 mM in 150 hours (~ 6 days), which is much slower than the first NO_3_^-^ injection (almost completely depleted after 5 days). On the other hand, the native microbial community still possesses potent NO_3_^-^ reducing capability after 64 days of starvation.

This figure shows that the reducing ability of NO_3_^-^ and NO_2_^-^ in the native microbial community is greatly inhibited due to cytotoxicity caused by NO_2_^-^ accumulation.


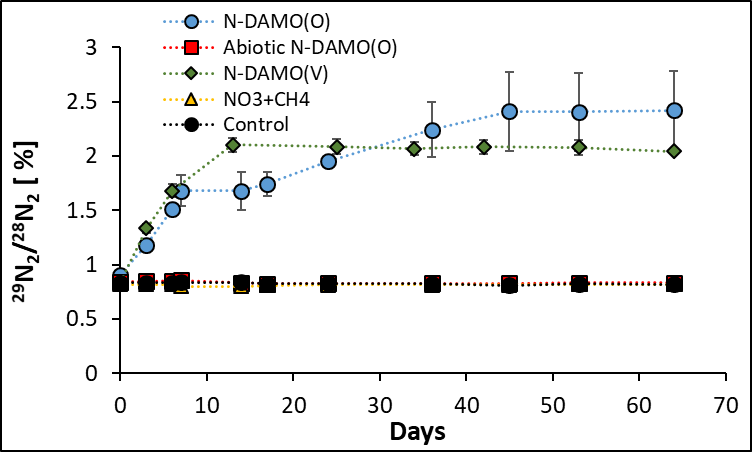


**Fig. S3 Changes in the ratio of ^29^N_2_ to ^28^N_2_ in the microcosms after 65 days of incubations.**


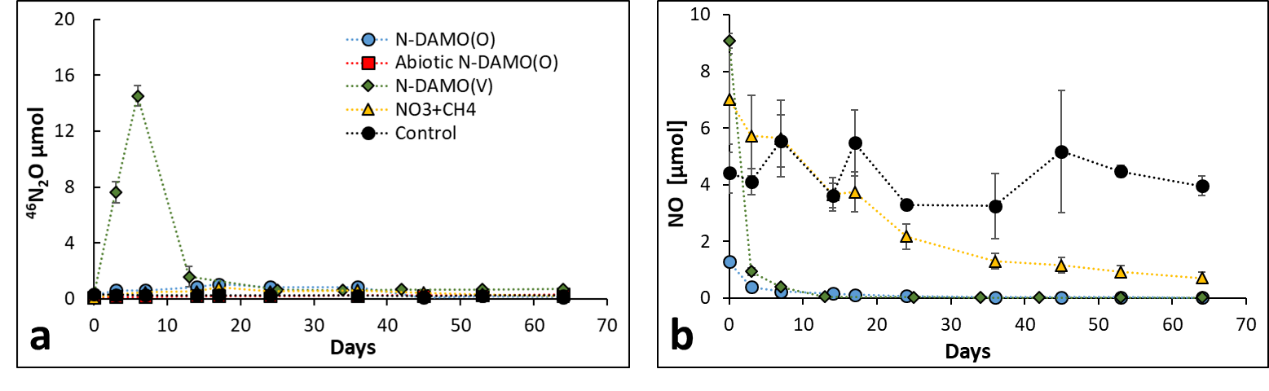


**Fig. S4 Changes in the contents of ^46^N_2_O a) and NO b) in the microcosms.** Only trace amounts of ^46^N_2_O were produced in the N-DAMO(V) microcosms and were quickly consumed, while in other treatments it remains at an extremely low level. The two N-DAMO enrichment cultures and native microbial communities exhibited the ability to consume NO, since the ^31^NO/^30^NO ratio was decreasing over time while the ^31^NO/^30^NO ratio remained relatively stable in the control. Overall, we believe that the primary reduction products are NH_4_^+^ and N_2_.


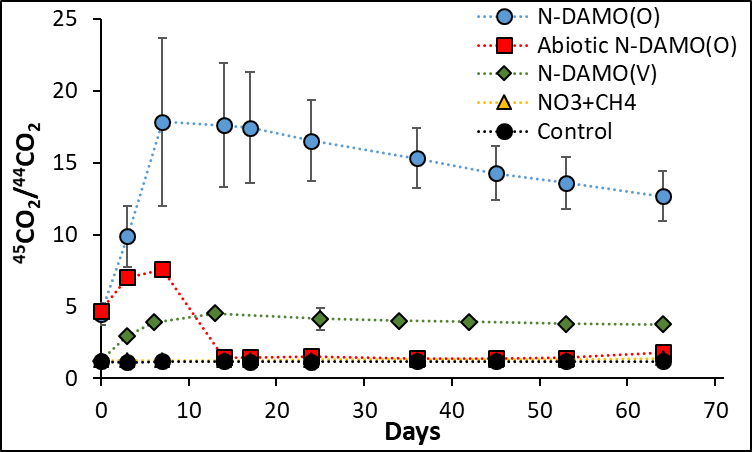


**Fig. S5 Changes in the content of ^45^CO_2_ in microcosms.** The content of ^45^CO_2_ increased sharply in two inoculated groups, reaching 43.5 mmol on day 7 and 36 mmol on day 13 then gradually decreased, whereas the ^13^CO_2_ content of the other three groups stay stable at around 3.5 mmol.


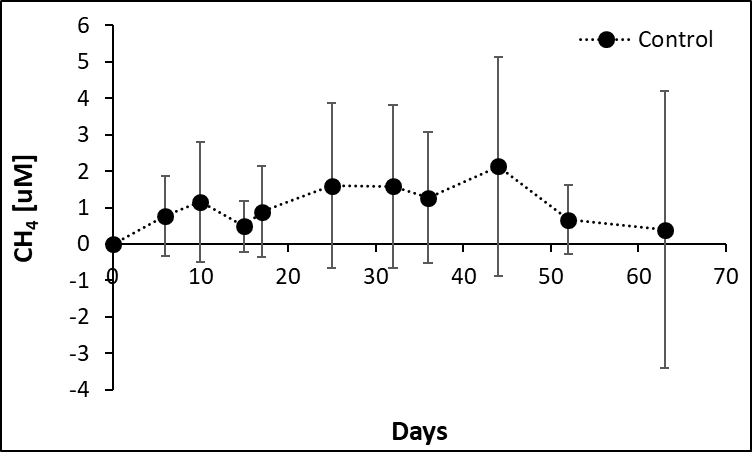


**Fig. S6 Changes in the concentration of CH_4_ in the control microcosms over the incubation time.**

**Calculation of the contribution of CH_4_ oxidation to NO_3_^-^ reduction.**

5CH_4_ + 8NO_3_^-^ + 8H^+^ → 5CO_2_ + 4N_2_ + 14H_2_O Equation S3

According to formula S3, one molecule of CH_4_ is oxidized, and the 1.6 NO_3_^-^ is reduced. Nitrate in the two N-DAMO inoculated groups was depleted in 10 days, and correspondingly, in N-DAMO(O) group, the CH_4_ level dropped from 0.75 mmol to 0.72 mmol, and in N-DAMO(V) group, it decreased from 0.90 to 0.86 mmol, in the first 10 days of incubation.

We assume that all CH_4_ consumption is due to the oxidation by NO_3_^-^. In the N-DAMO(O) group, where 30 μmol of CH_4_ was oxidized, representing 48 μmol of NO_3_^-^ was reduced to N_2_ or NH_4_^+^. Similarly, in N-DAMO(V) group, 40 μmol of CH_4_ was consumed, meaning 64 μmol of NO_3_^-^ was reduced. At the beginning of the incubation, there were 310 μmol of NO_3_^-^ in the microcosms (5 mM*62 mL). From this, we can calculate that about 15.48% of total NO_3_^-^ reduction is attributed to CH_4_ oxidation, whereas in N-DAMO(V) group this is 20.65%.

NO_3_^-^ + CH_4_ + 2H^+^ → CO_2_ + NH_4_^+^ + H_2_O Equation S4

In addition, according to formula S4, we also observed that N-DAMO enrichment culture undergoes the NDRA process, so for every molecule of CH_4_ consumed, only one molecule of NO_3_^-^ is reduced to NH_4_^+^. In microcosms, the denitrification and DNRA processes were carried out simultaneously, and we do not know the contribution ratio of the two. We can speculate that the contribution of N-DAMO to the total NO_3_^-^ reduction in microcosms is lower than 20%, and the rest of the NO_3_^-^ reduction was caused by oxidation of DOM.
